# Molecular subtyping of esophageal squamous cell carcinoma by large-scale transcriptional profiling: Characterization, therapeutic targets, and prognostic value

**DOI:** 10.3389/fgene.2022.1033214

**Published:** 2022-11-08

**Authors:** Danke Wang, Jiacheng Dai, Chen Suo, Shangzi Wang, Yuting Zhang, Xingdong Chen

**Affiliations:** ^1^ State Key Laboratory of Genetic Engineering, Human Phenome Institute, School of Life Sciences, Fudan University, Shanghai, China; ^2^ Fudan University Taizhou Institute of Health Sciences, Taizhou, China; ^3^ Department of Epidemiology, School of Public Health, Fudan University, Shanghai, China; ^4^ Shanghai Institute of Infectious Disease and Biosecurity, Shanghai, China; ^5^ Department of Neurology, Huashan Hospital, Fudan University, Shanghai, China; ^6^ Yiwu Research Institute of Fudan University, Yiwu, Zhejiang, China

**Keywords:** ESCC, gene expression profile, subtype, integrate, GSEA, WGCNA

## Abstract

The tumor heterogeneity of the transcriptional profiles is independent of genetic variation. Several studies have successfully identified esophageal squamous cell carcinoma (ESCC) subtypes based on the somatic mutation profile and copy number variations on the genome. However, transcriptome-based classification is limited. In this study, we classified 141 patients with ESCC into three subtypes (Subtype 1, Subtype 2, and Subtype 3) *via* tumor sample gene expression profiling. Differential gene expression (DGE) analysis of paired tumor and normal samples for each subtype revealed significant difference among subtypes. Moreover, the degree of change in the expression levels of most genes gradually increased from Subtype 1 to Subtype 3. Gene set enrichment analysis (GSEA) identified the representative pathways in each subtype: Subtype 1, abnormal Wnt signaling pathway activation; Subtype 2, inhibition of glycogen metabolism; and Subtype 3, downregulation of neutrophil degranulation process. Weighted gene co-expression network analysis (WGCNA) was used to elucidate the finer regulation of biological pathways and discover hub genes. Subsequently, nine hub genes (*CORO1A, CD180, SASH3, CD52, CD300A, CD14, DUSP1, KIF14, and MCM2*) were validated to be associated with survival in ESCC based on the RNA sequencing (RNA-seq) data from The Cancer Genome Atlas (TCGA) database. The clustering analysis of ESCC granted better understanding of the molecular characteristics of ESCC and led to the discover of new potential therapeutic targets that may contribute to the clinical treatment of ESCC.

## 1 Introduction

In 2020, esophageal carcinoma (EC) was the seventh most common cancer worldwide with 604,000 new cases, contributing 3.1% of the total new cancer cases, and was ranked sixth in mortality worldwide (544,000 deaths) (Sung et al., 2021). Esophageal squamous cell carcinoma (ESCC) and esophageal adenocarcinoma (EAC) are the two main EC subtypes (Siewert and Ott, 2007; Della Guardia et al., 2022), with ESCC counting for approximately 90% of EC cases worldwide (Smyth et al., 2017). The development of next-generation sequencing technologies has yielded a deeper understanding of ESCC genomic features *via* sequencing and analysis the genomes and transcriptomes of millions of patients with ESCC. The analyses revealed that ESCC has extensive inter- and intra-tumor heterogeneity (Hu et al., 2009; Lin et al., 2018).

Regarding tumor heterogeneity, several studies (Liu et al., 2016; Cancer Genome Atlas Research Network et al., 2017) have identified ESCC subtypes based on the somatic mutation profile and copy number variation on the genome. However, recent single-cell RNA sequencing (RNA-seq) studies have demonstrated that cancer cell state heterogeneity is largely independent of genetic variation (Halbritter et al., 2019; Guo et al., 2020; LaFave et al., 2020; Marjanovic et al., 2020). The transcriptional landscape is reprogrammed with cancer progression, metastasis, and therapy resistance (Hanahan and Weinberg, 2011; Quintanal-Villalonga et al., 2020). Therefore, identifying ESCC subtypes based on the gene expression profile of the tumor sample can reveal some molecular features that cannot be detected in genome-based classification. There are many successful applications for identifying tumor subtypes based on the gene expression profile, including that for colon cancer (Marisa et al., 2013; Guinney et al., 2015), non-small cell lung cancer (Chen et al., 2017) and uterine leiomyosarcoma (An et al., 2017). These transcriptional profile-based classification studies revealed clinically valuable targets. Attempts have also been made to classify ESCC based on its gene expression profile. Wang et al. (Wang et al., 2019) have classified Asian patients with ESCC into two subtypes; the selected genes were clustered and only genes with large standard deviations in the ESCC cohort were selected. Nevertheless, classification based on selected genes may bias the result, neglecting genes that are less varied but that are important in overall regulation. Therefore, it is necessary to characterize ESCC subtypes considering an unbiased transcriptome level.

In this study, we classified 141 patients with ESCC into three subtypes based on the gene expression profile of the patients’ tumor samples. The differences in individual gene expression level changes among the three subtypes were identified with differential gene expression (DGE) analysis. Gene set enrichment analysis (GSEA) and weighted gene co-expression network analysis (WGCNA) were used to explore how the gene expression levels co-varied together. *Via* this analysis series, we clearly described the molecular characteristics of each subtype. We discovered important genes and the biological pathways that may affect ESCC prognosis. Our study provides an in-depth understanding of ESCC molecular features and demonstrates potential targets for ESCC clinical treatment.

## 2 Materials and methods

### 2.1 Data collection and quality control

The raw microarray gene expression data from 141 ESCC patient tumors and the paired normal samples across seven datasets were obtained from the Gene Expression Omnibus (GEO). The dataset inclusion criteria were: 1) gene expression data from paired tumor and normal tissue samples were available; 2) the patients had not undergone previous treatment. The following datasets were included in this study: GSE17351, GSE20347, GSE23400, GSE38129, GSE77861, GSE161533, and GSE100942 (Table 1) (Hu et al., 2010; Lee et al., 2010; Su et al., 2011; Hu et al., 2015; Erkizan et al., 2017; Ming et al., 2018). Among the seven datasets, the samples in GSE77861 were obtained from African American patients and samples in the remaining six datasets were from Asian patients.

**TABLE 1:** Information of the GEO datasets involved in this study.

GEO accession	Platforms	Sample size	Race	PMID
GSE17351	GPL570	10	Japanese	20042640
GSE20347	GPL571	34	Chinese	20955586
GSE23400	GPL96	106	Chinese	21385931
GSE38129	GPL571	60	Chinese	26409826
GDE77861	GPL570	14	African American	28629367
GSE161533	GPL570	56	Chinese	—
GSE100942	GPL570	8	Chinese	29290801

The Affymetrix microarray data were robust multiarray averaging (RMA) normalized (background processing, log2 transformation, quantile scaling, and probe expression measurement) in the R package affy (Gautier et al., 2004). Then, all available biological and technical covariates except for the diagnostic group were regressed from each individual expression dataset. After the above preprocessing had been performed on each dataset, all seven datasets were merged. The batch effect was corrected with the ComBat function of the R sva package (Leek et al., 2012). Outliers were identified with the principal component analysis (PCA) in the R package FactoMineR.

The RNA-seq data of tumor tissue samples from patients with ESCC were downloaded from The Cancer Genome Atlas (TCGA) database using the R package TCGAbiolinks (Colaprico et al., 2016). The screening and elimination yielded the gene expression profiling data of tumor tissues from 80 patients. The TPM (transcripts per million) data that normalized gene length and sequencing depth were used for subsequent analysis.

### 2.2 Clustering

The function hclust was used to hierarchical clustering the 141 ESCC tumor samples through gene expression profiling. We used the Euclidean method to calculate the Euclidean distance between samples and the ward.D method to cluster the 141 samples. The parameters of the clustering based on the K-means method were centers = 3, nstart = 25.

### 2.3 Differential gene expression analysis

For each subtype, DGE analysis was performed between paired tumor and normal samples, using the R package limma (Ritchie et al., 2015). The differential expression genes (DEGs) were identified as adj.P.Val cutoff <0.05 (Benjamini Hochberg false discovery rate (FDR) correction) (Ge et al., 2021) (FDR <0.05) and |log_2_ fold change (log_2_FC)| > 1.

### 2.4 Gene set enrichment analysis and immune cell infiltration analysis

GSEA (Subramanian et al., 2005) was performed with the function gseGO in the R package clusterProfiler (Yu et al., 2012) based on the Gene Ontology (GO) database. GSEA was performed on the three ESCC subtypes and the log_2_FC of each gene was used as the basis for the gene ranking. Significantly enriched pathways (FDR <0.05) were available.

The gene expression profile data of the 141 ESCC tumor samples were combined for immune cell infiltration analysis using single-sample GSEA (ssGSEA) in R package GSVA (Hanzelmann et al., 2013). The immune cell marker genes constituted the background gene set for the immune infiltration analysis (Charoentong et al., 2017).

### 2.5 Weighted gene co-expression network analysis

Network analysis was performed with the WGCNA package (Langfelder and Horvath, 2008) in R. Approximate scale-free topology (*R* ^2^ > 0.85) was achieved using a soft threshold power of 7. The network was constructed using all 13,515 gene expression profiling data from the 141 paired ESCC tumor and normal samples. The other network construction parameters were maxBlockSize = 5,000, minModuleSize = 30, TOMType = “unsigned”, reassignThreshold = 0, and mergeCutHeight = 0.25.

### 2.6 Enrichment analysis

GO enrichment analysis was performed with the function enrichGO in the R package clusterProfiler based on the GO database. The significantly enriched pathways had FDR <0.05.

Cell type enrichment analysis was performed based on the marker genes (Xu et al., 2021) of different cell types. Significantly enriched cell types were obtained with *p* < 0.05 (Fisher’s exact test).

### 2.7 Survival analysis

Survival analysis was performed with the RNA-seq data of 80 ESCC tumor tissue samples from TCGA database. Survival analysis and survival curve plotting were performed using the R packages survival and survminer, respectively. For each gene among the 80 samples, samples with expression levels higher than the median value were defined as the high-expression group and those below the median value were defined as the low-expression group.

## 3 Results

### 3.1 Characteristics of the mRNA microarray data and analysis pipeline

**FIGURE 1:**
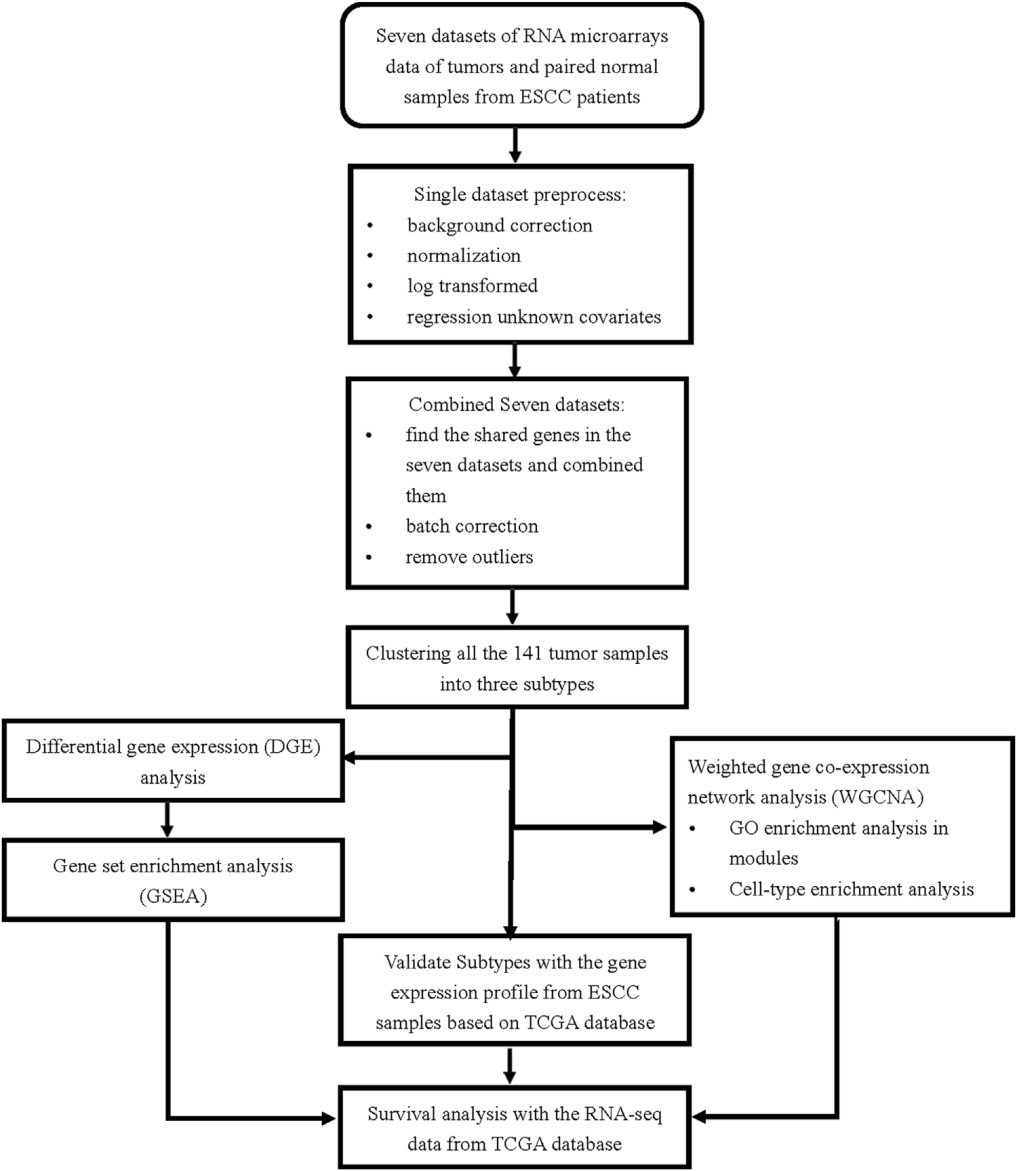
The pipeline of this analysis.

The analysis pipeline is depicted in Figure 1. After strict data preprocessing, the seven ESCC mRNA microarray datasets were merged, and batch effects were corrected using the function ComBat. PCA was performed with batches as groups before and after batch correction. There was a large distance between the datasets before batch correction (Supplementary Figure S1A), and the data distribution was uniform after batch correction (Supplementary Figure S1B). The boxplots of samples grouped by batch before and after batch correction also reflected this change (Supplementary Figures S1C, D). The results suggested that the batch effects among the seven datasets were eliminated. We used the combined dataset for subsequent analysis.

When PCA was performed based on tumor and normal grouping, three normal samples which were abnormally grouped with the tumor samples (Supplementary Figure S1E). Therefore, we removed these three samples, which included the match normal (GSM573851, GSM573889, and GSM573852) and tumor samples (GSM573904, GSM573942, and GSM573905). The remaining 282 samples were divided into tumor and normal groups (Supplementary Figure S1F), a distinct transcriptomic pattern was indicated between the two groups.

The 13,515-gene expression profile data of the 141 paired ESCC tumor and normal samples were included in the study. The ESCC subtypes were identified using the gene expression profiles of the 141 ESCC tumor samples. The analyses mainly included: 1) hierarchical clustering of the 141 ESCC tumor sample; 2) DGE analysis of paired tumor and normal samples of each subtype to determine gene expression level changes; 3) GSEA and WGCNA to identify biological pathway regulation and discover hub genes; 4) survival analysis of the genes found in 3) based on the RNA-seq data from 80 ESCC tumor samples in the TCGA database; 5) validation of the subtypes found in 1) using the TCGA RNA-seq data.

### 3.2 The results of clustering

**FIGURE 2:**
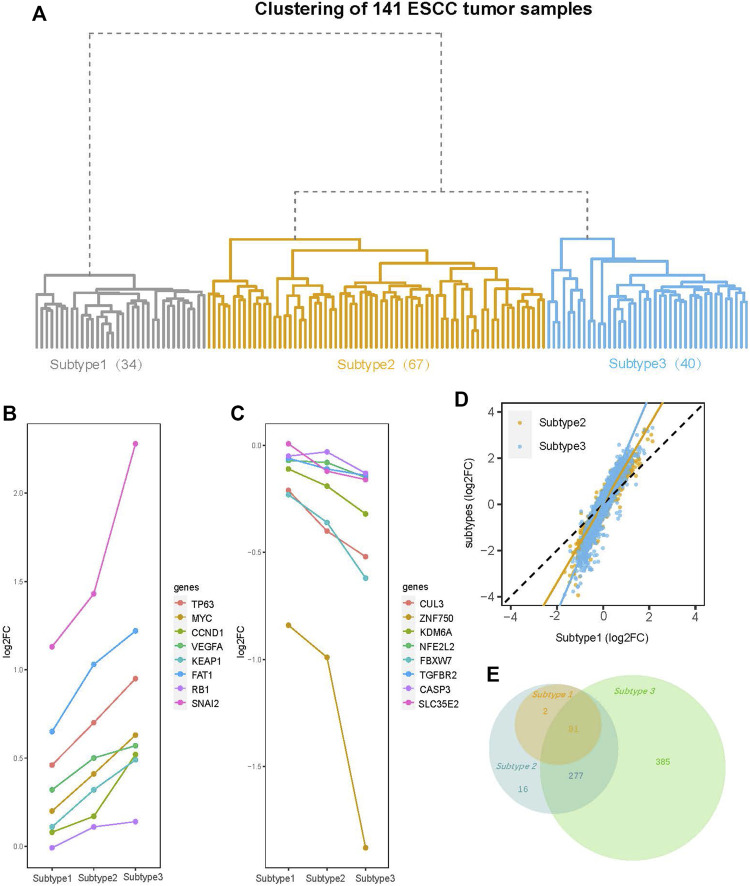
Clustering tree of the 141 ESCC tumor samples and an overview of differential gene expression (DGE) analysis. **(A)** The hierarchical clustering tree diagram of 141 ESCC tumor samples constructed by the gene expression profile. The 141 ESCC tumor samples were clustered into three subtypes (Subtype 1, Subtype 2, and Subtype 3), with sample sizes of 34, 67, and 40 for Subtype 1 to Subtype 3, respectively. **(B)** The log_2_ fold-change (log_2_FC) of the genes (TP63, MYC, CCND1, VEGFA, KEAP1, FAT1, RB1, and SNAI2) with amplification mutations on ESCC genome are increased gradually from Subtype 1 to Subtype 3. **(C)** The log_2_FC of ESCC genomic insertion or deletion mutant genes (CUL3, ZNF750, KDM6A, NFE2L2, FBXW7, TGFBR2 CASP3, and SLC35E2) decreased gradually from Subtype 1 to Subtype 3. **(D)** Comparison of log_2_FC of all the 13515 genes between subtypes. Among three subtypes, the degree of change in the expression levels of most genes is: Subtype 3 > Subtype 2 > Subtype 1. **(E)** Venn diagram shows overlaps of differential expression genes (DEGs) (FDR <0.05, |log_2_FC| > 1) among three subtypes. The numbers of DEGs include both up- and down-regulated genes in tumor tissue compared to normal tissue.

Hierarchical clustering of the 141 ESCC tumor sample transcriptome profiles revealed a total of three subtypes, which we designated Subtype 1 (34 samples), Subtype 2 (67 samples), and Subtype 3 (40 samples). Subtype 1 was under an independent branch of the clustering tree and Subtypes 2 and 3 were two subgroups under the same branch (Figure 2A).

To further confirm that we have eliminated batch effects, we recolored the hierarchical clustering tree using batch as color label. The result showed that the samples from each study were distributed across the three subtypes (Supplementary Figure S2A). So, the subtypes are not driven by different studies.

To explore whether our subtyping is sensitive to the clustering method, we re-clustered the 141 samples into three subtypes (named as K-means_Subtype1, K-means_Subtype2, and K-means_Subtype3) using the K-means method. The result showed that the three subtypes clustered by the K-means method were consistent with the subtypes obtained by the hierarchical clustering in more than 80% of the samples (Figure 2A, Supplementary Figure S2B). So, it can be concluded that the subtypes we obtained are not sensitive to the clustering method.

### 3.3 Results of differential gene expression analysis

DGE analysis was performed on the paired tumor and normal samples for each subtype. We first focused on the most commonly mutated genes in ESCC. The log_2_FC of these genes exhibited a tendency change from Subtype 1 to Subtype 3. For example, the log_2_FC of genes in the ESCC genome with amplification mutations, such as *MYC, TP63, CCND1, VEGFA,* and *SNAI2* (Song et al., 2014; Liu et al., 2016; Sawada et al., 2016; Cancer Genome Atlas Research Network et al., 2017), increased gradually from Subtype 1 to Subtype 3 (Figure 2B). The log_2_FC of ESCC genomic insertion or deletion mutant genes [*CUL3, ZNF750, KDM6A, NFE2L2, and SLC35E2* (Song et al., 2014; Liu et al., 2016; Sawada et al., 2016; Cancer Genome Atlas Research Network et al., 2017)] decreased gradually from Subtype 1 to Subtype 3 (Figure 2C). Viewing of the total 13,515 genes, the degree of change in the expression levels of most genes gradually increased from Subtype 1 to Subtype 3 (Figure 2D).

The DEG numbers were greatly different across the three subtypes. Under the thresholds (FDR <0.05; log_2_FC ≥ |1|), Subtypes 1, 2, and 3 had 83, 376, and 743 total DEGs, respectively (Supplementary Figure S3). The dramatic increase in the DEG number from Subtype 1 to Subtype 3 suggested striking differences among the subtypes even if they were identified as the same cancer type. Further exploration of the relationship between the DEGs of the three subtypes revealed that the Subtype 2 DEGs included all Subtype 1 DEGs, while the Subtype 3 DEGs did not completely encompass the Subtypes 1 and 2 DEGs (Figure 2E). Eighteen DEGs were specific to Subtype 1 or 2 rather than Subtype 3 (Figure 2E, Supplementary Figure S4A). The 16 upregulated DEGs among these 18 genes are enriched in the immune-related pathways (Supplementary Figure S4B).

### 3.4 Results of gene set enrichment analysis and single-sample GSEA

**FIGURE 3:**
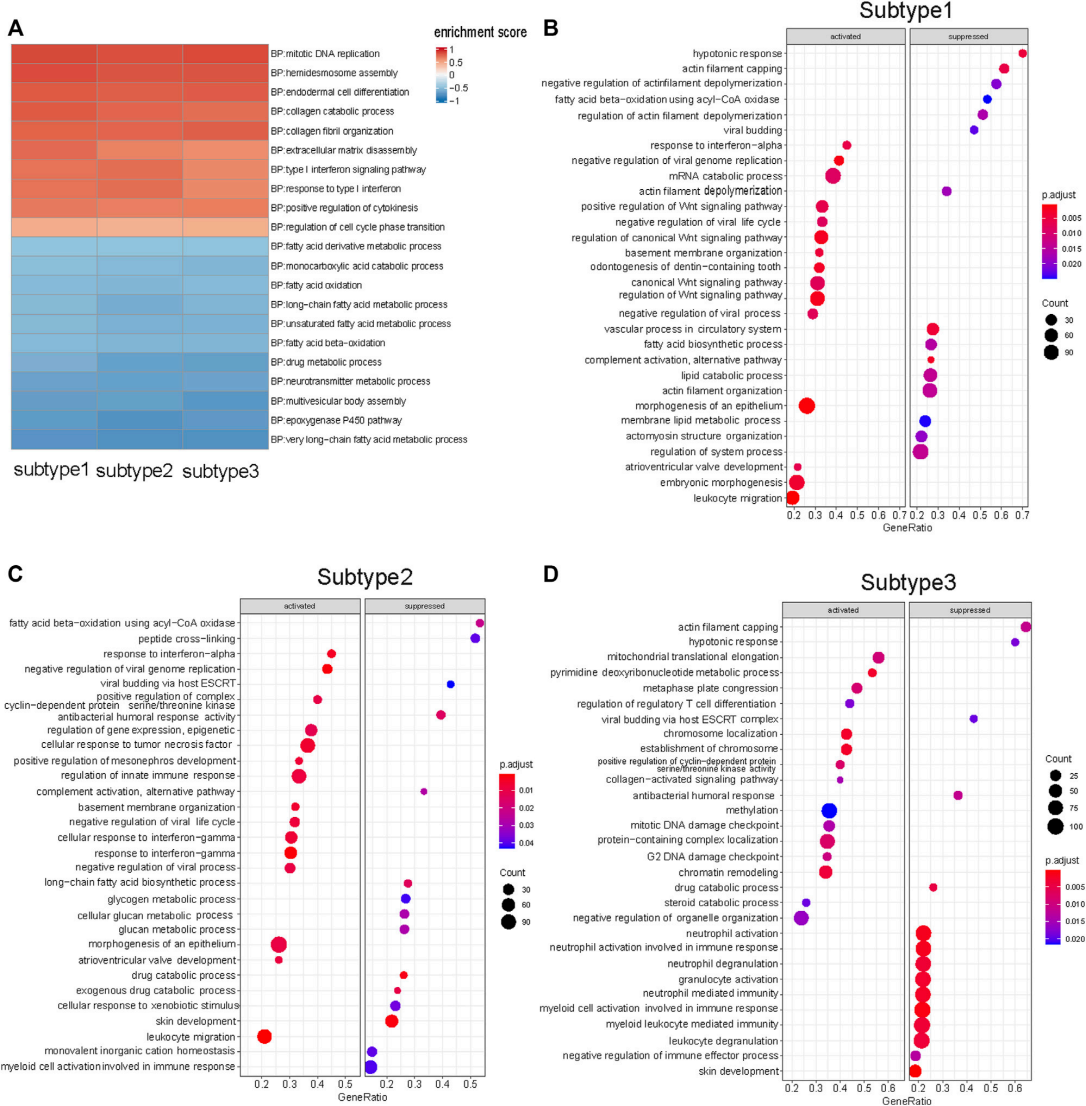
GSEA results of three subtypes. **(A)** Ten up-regulated and ten down-regulated biological pathways were selected from the shared enriched pathways in three subtypes tumor samples. **(B)** Top 15 biological pathways that specific up- and down-regulated in Subtype 1 tumor samples. Wnt signaling pathway was significantly up-regulated in Subtype 1. **(C)** Top 15 biological pathways that specific up- and down-regulated in Subtype 2 tumor samples. Glycogen metabolism biological pathway was significantly down-regulated in Subtype 2. **(D)** Top 15 biological pathways that specific up- and down-regulated in Subtype 3 tumor samples. Neutrophil-mediated immunological pathways were down-regulated in Subtype 3. (FDR <0.05).

In this study, we used GSEA to determine whether a set of genes involved in a biological pathway demonstrated statistically significant differences between tumor and normal status (Subramanian et al., 2005). GSEA of each subtype enabled the discovery of how various biological pathways were regulated in each subtype. The biological pathways commonly upregulated in the three subtypes were those for chromosomal DNA replication, endodermal cell differentiation, and collagen fibril organization (Figure 3A) (Supplementary Material S1). The commonly downregulated biological pathways included those for fatty acid oxidation metabolism, and keratinocyte differentiation (Figure 3A) (Supplementary Material S1). The shared biological pathway regulation among the three subtypes was consistent with the findings of previous ESCC studies. (Su et al., 2011; Erkizan et al., 2017).

There were representative enriched pathways in tumor samples of each subtype. Subtype 1 was characterized by significant Wnt signaling pathway upregulation (FDR <0.05) (Figure 3B). Glycogen metabolism downregulation was the hallmark of Subtype 2 (Figure 3C). Subtype 3 featured markedly inhibited neutrophil-mediated immunological pathways (FDR <0.05), in which downregulated neutrophil degranulation was the primary manifestation (Figure 3D).

The degree of immune cell infiltration in tumor samples from the three subtypes was assessed with ssGSEA. The results revealed fewer infiltrating T cells in Subtype 1 tumor samples as compared to those of Subtypes 2 and 3 and less neutrophil infiltration in Subtype 3 tumor tissues as compared with that of Subtypes 1 and 2 (Supplementary Figure S5).

### 3.5 Enrichment results of modules that constructed by weighted gene co-expression network analysis

We performed WGCNA to characterize the involved biological pathways more specifically. The genes regulated in the same pattern were clustered in one co-expression module based on the correlation coefficient weighted value. This approach fully accounted for the genes that change little but that may be important in overall regulation. All gene expression profiles of the 141 paired ESCC tumor and normal samples were considered in the co-expression network construction, which included a total of 14 modules, including module 0 (genes with irregular expression) (Supplementary Material S2).

**FIGURE 4:**
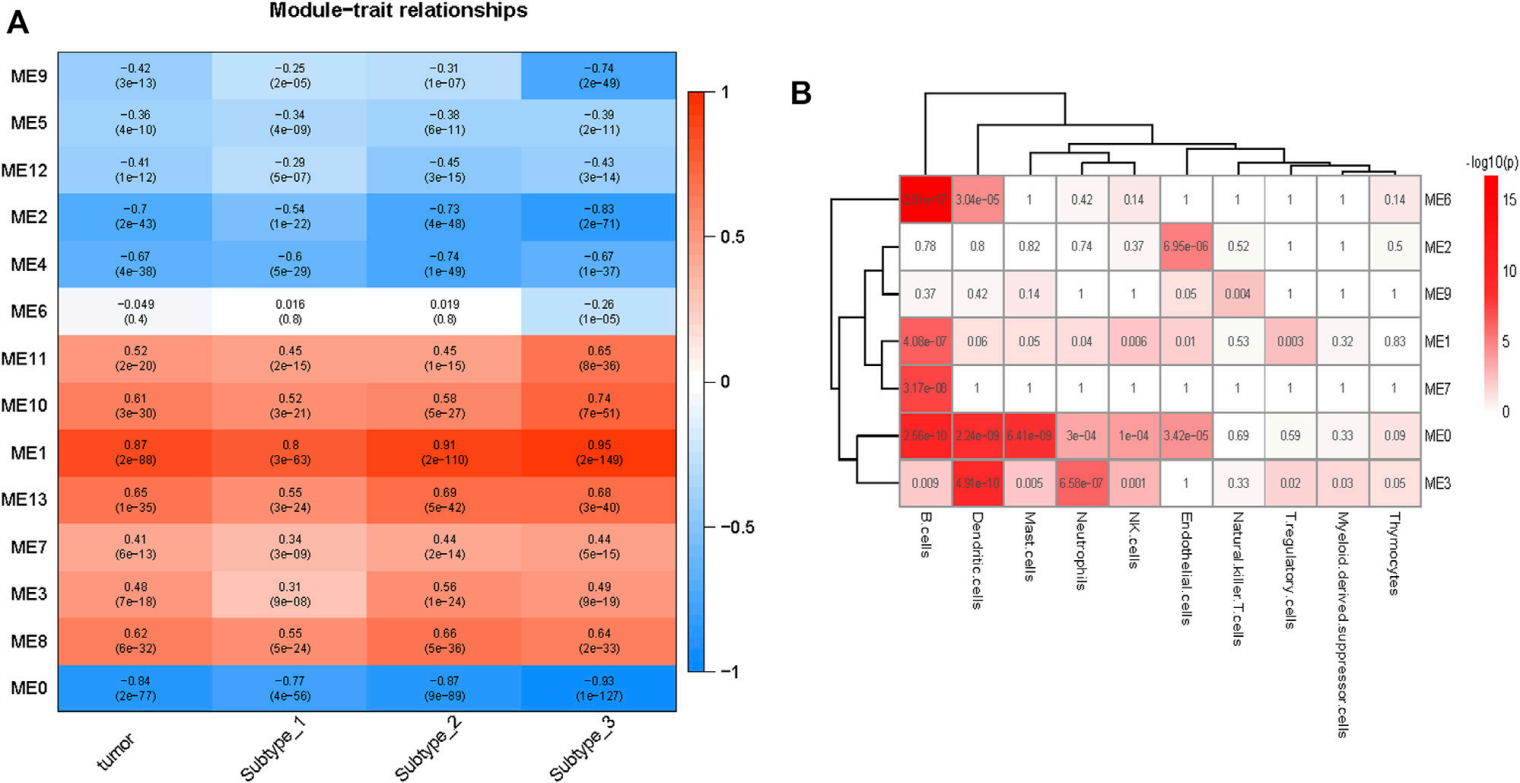
Overview of the modules constructed by WGCNA. **(A)** Correlations of modules to tumor. Modules 1, 3, 7, 8, 10,11, and 13 are positive correlated with tumor and modules 0, 2, 4, 5, 9, and 12 are negative correlated with tumor. Module 6 was negatively correlated with Subtype 3 only. **(B)** Enrichment of various cells in different modules (module0, 1, 2, 3, 6, 7, and 9), the *p* values (Fisher’s exact test) are shown. B cells were enriched in modules 1, 3, and 6, the endothelial cells were enriched in modules 2 and 9.

The correlations between the modules and tumor are depicted in Figure 4A. Seven (modules 1, 3, 7, 8, 10, 11, and 13) and six (modules 0, 2, 4, 5, 9, and 12) modules were positively and negatively correlated with tumor, respectively (FDR <0.05). From Subtype 1 to Subtype 3, eight modules (module 0, 1, 2, 5, 7, 9, 10, and 11) demonstrated a gradually stronger correlation between modules and subtypes, and five modules (module 3, 4, 8, 12, and 13) presented stronger correlations from Subtype 1 to Subtype 2 and weakened correlations from Subtype 2 to Subtype 3. Module 6 was highly distinctive, demonstrating no significant correlation with Subtype 1 or 2, but demonstrating a remarkable negative correlation (r = −0.26, p = 1E-05) with Subtype 3.

**FIGURE 5:**
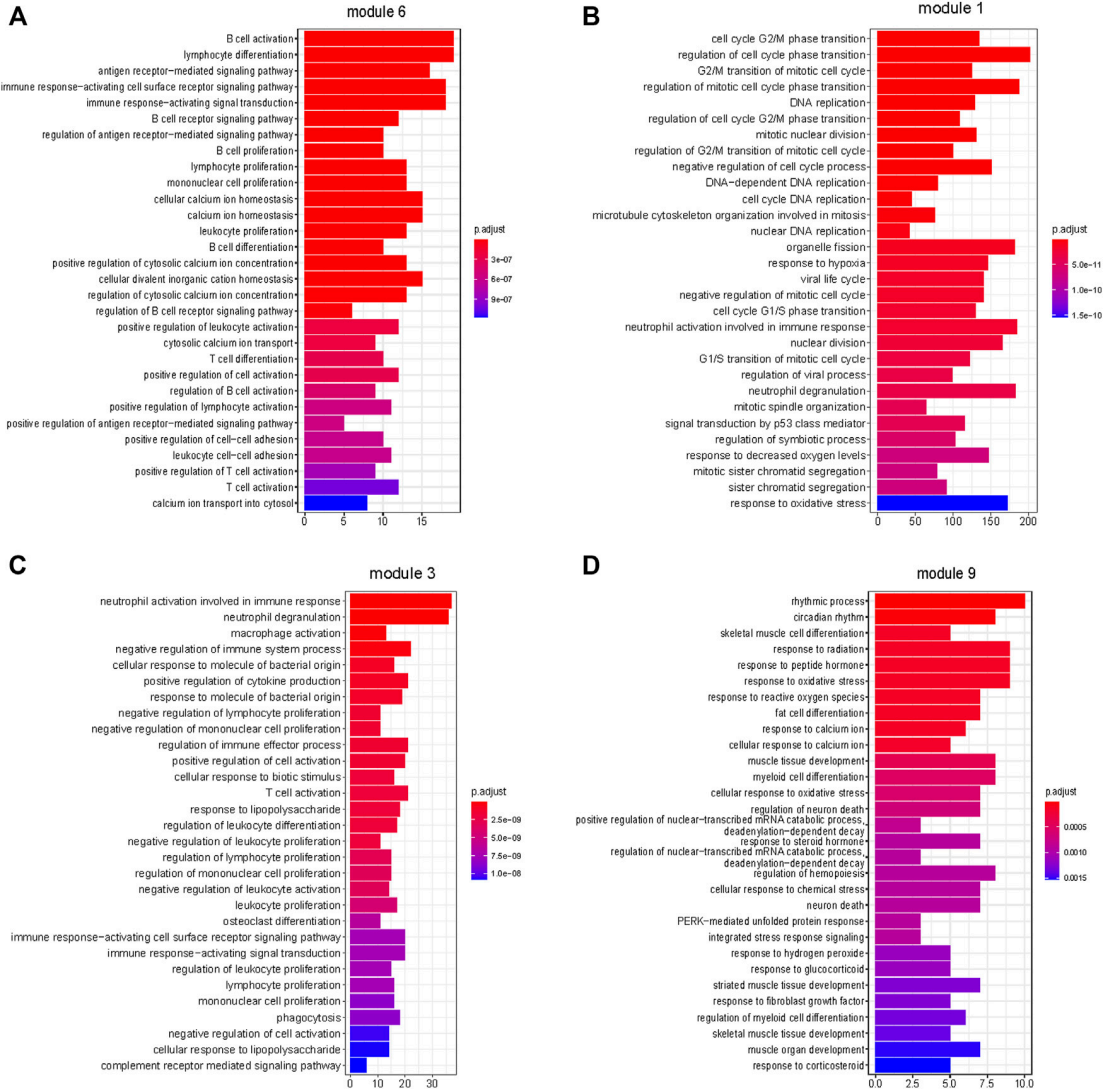
Enrichment of biological pathways in modules. **(A–D)** The top 30 pathways that enriched in module 6, module 1, module 3, and module 9, respectively. **(A,C)** Immune-related processes were enriched in modules 6 and 3. **(B)** Cell proliferation-related processes were enriched in module 1. **(D)** Cellular responses to external stimuli were enriched in module 9. (FDR <0.05).

Module 6 were enriched in a broad range of immune-related pathways. The biological pathways enriched in module 6 included B cell activation, T cell differentiation, lymphocyte differentiation and calcium homeostasis (FDR <0.05) (Figure 5A). The cell type enrichment analysis revealed that B cells and dendritic cells were enriched in module 6 (FDR <0.05) (Figure 4B).

Based on the above results, modules 0, 1, and 3 of neutrophil enrichment were the modules of interest (Figure 4B). In modules 1 and 3 (both were positively correlated with the three subtypes), the neutrophil degranulation process was among the top 30 enriched pathways (Figures 5B, C) (FDR <0.05). In addition, module 1 enriches cell cycle-related biological processes, and module 3 also enriches other immune-related biological pathways. The neutrophil chemotaxis pathway was enriched in module 0 (FDR <0.05) (Supplementary Material S3), which was negatively correlated with the three subtypes (correlation between module 0 and Subtype 3: r = −0.93, p = 1E-127).

Endothelial cells were enriched in modules 2 and 9 (*p* ≤ 0.05) (Figure 4B), both of which were negatively correlated with tumor. The negative correlation increased gradually from Subtype 1 to Subtype 3. Biological pathways such as cellular rhythm, response to radiation, and response to oxidative stress were enriched in module 9 (Figure 5D) while, the muscle contraction and myofibril assembly biological pathways were enriched in module 2 (Supplementary Figure S6). We were able to conclude that endothelial cell contractility and responsiveness to external stimuli are affected in ESCC.

### 3.6 Results of survival analysis

**FIGURE 6:**
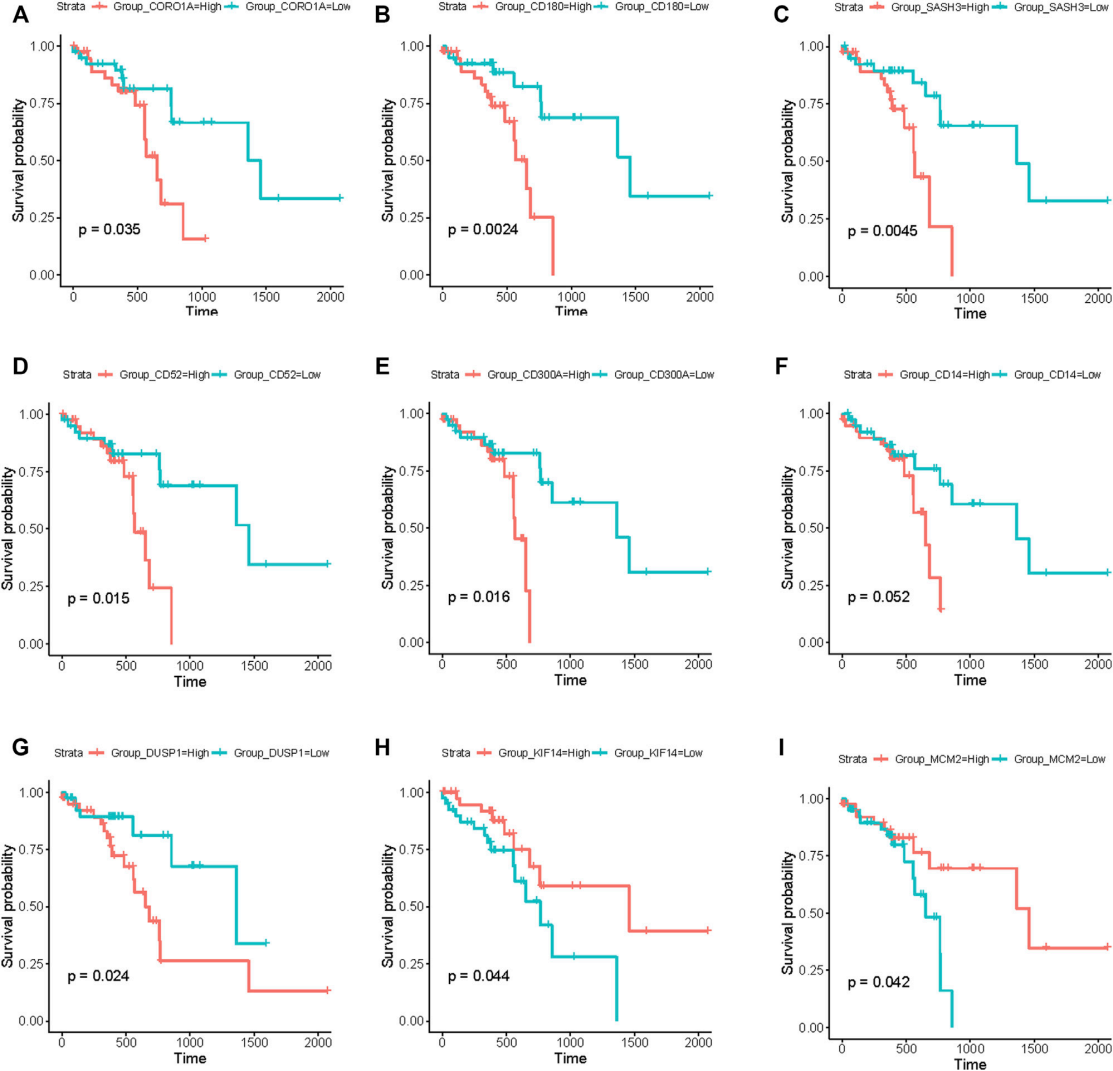
Survival analysis using the RNA-seq data from TCGA database. **(A–I)** Kaplan–Meier curves showing that the expression levels of these nine genes (CORO1A, CD180, SASH3, CD52, CD300A, CD14, DUSP1, KIF14, and MCM2) were significantly associated with survival of patients with ESCC. **(A–G)** Seven genes (CORO1A, CD180, SASH3, CD52, CD300A, CD14, and DUSP1) were negatively correlated with survival. **(H,I)** Two genes (KIF14 and MCM2) were positively correlated with survival. (*p* < 0.05).

Based on the above module enrichment results, we performed survival analysis of nine genes (*CORO1A*, *CD180*, *SASH3*, *CD52*, *CD300A*, *CD14*, *DUSP1*, *KIF14*, and *MCM2*) from the hub genes in these modules of interest and the genes involved in biological pathways that are important in tumor progression. We validated these genes by TCGA dataset survival analysis. The high levels of seven genes (CORO1A, CD180, SASH3, CD52, CD300A, CD14, and DUSP1) were related with poor survival in ESCC (Figures 6A–G) and KIF14 and MCM2 expression levels were positively correlated with better survival (Figures 6H, I) (*p* < 0.05). *CORO1A, CD180, SASH3,* and *CD52* were located in module 6, *CD300A* and *CD14* were involved in module 3, module 9 contained *DUSP1*, module 1 contained *KIF14,* and *MCM2* (Table 2).

**TABLE 2:** The profile of the genes that correlated with survival of ESCC patients.

Gene	Module	Correlation with survival	Gene ontology enrichment
CORO1A	Module6	negative	lymphocyte proliferation
			cellular calcium ion homeostasis
			leukocyte proliferation
CD180	Module6	negative	B cell activation
			B cell proliferation
SASH3	Module6	negative	B cell activation; lymphocyte differentiation
CD52	Module6	negative	cellular calcium ion homeostasis
			calcium ion homeostasis
			positive regulation of cytosolic calcium ion concentration
CD300A	Module3	negative	neutrophil activation involved in immune response
			neutrophil degranulation
CD14	Module3	negative	neutrophil activation involved in immune response
			neutrophil degranulation
			cellular response to molecule of bacterial origin
DUSP1	Module9	negative	response to radiation
			response to oxidative stress
KIF14	Module1	positive	cell cycle G2/M phase transition
			regulation of cell cycle phase transition
MCM2	Module1	positive	DNA replication
			DNA-dependent DNA replication

### 3.7 Expression levels of the survival-related genes in the three subtypes

**FIGURE 7:**
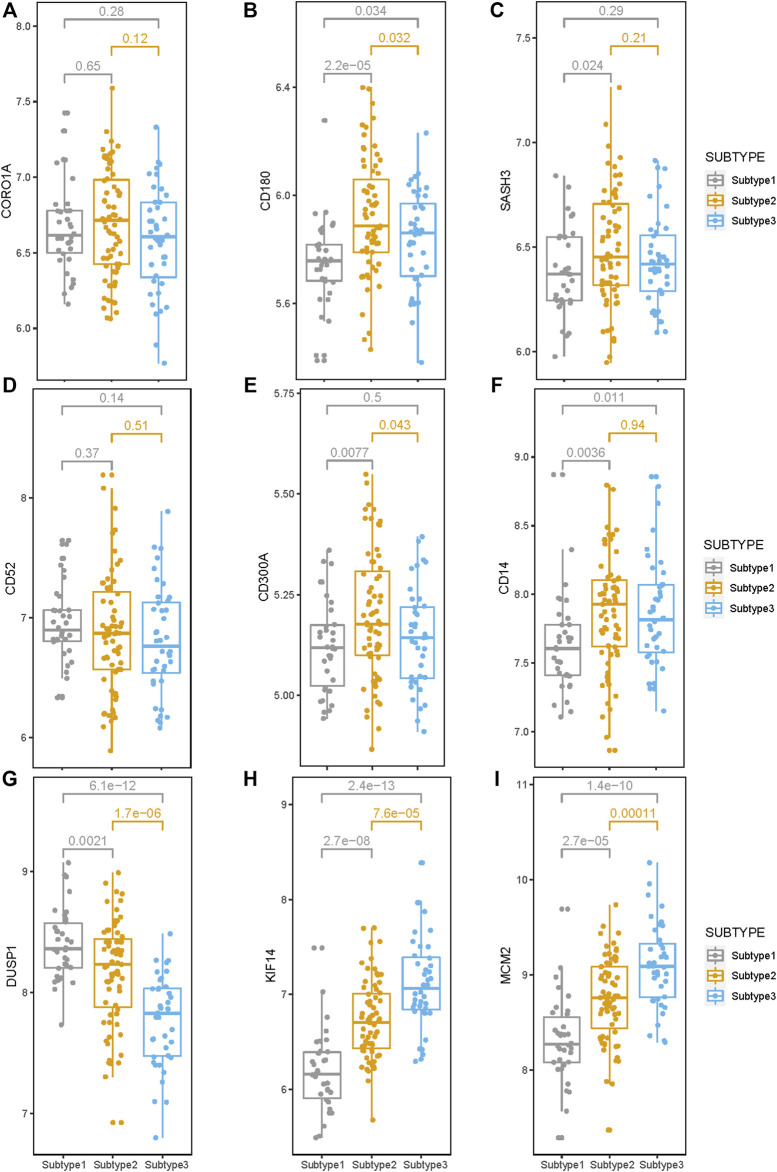
Expression levels of the nine survival-related genes in the three subtypes. **(A–I)** The expression level of the nine survival-related genes in the three subtypes. Expression levels of these seven (CD180, SASH3, CD300A, CD14, DUSP1, KIF14, and MCM2) survival-related genes are significantly different among these three subtypes. (*p* < 0.05).

The expression levels of the nine survival-related genes in the three subtypes may indicate the survival of subtypes to a certain extent. The gene expression levels of four genes (CD180, SASH3, CD300A, and CD14) inversely associated with survival were significantly higher in Subtype 2 or 3 than in Subtype 1 (Figures 7B, C, E, F). The four genes are involved in the immune-related pathways. So, tumor immunity may be an important factor affecting survival time in Subtype 2 or 3.

Compared with Subtypes 2 and 3, *DUSP1* that negatively correlated with survival, has higher expression in Subtype 1 (Figure 7G), while *KIF14* and *MCM2*, which are positively correlated with survival, have lower expression in Subtype 1 (Figures 7H, I). Therefore, the biological pathways that respond to various cellular stress conditions and the DNA replication pathways play important roles in Subtype 1.

### 3.8 Validation of the generality of esophageal squamous cell carcinoma subtypes

**FIGURE 8:**
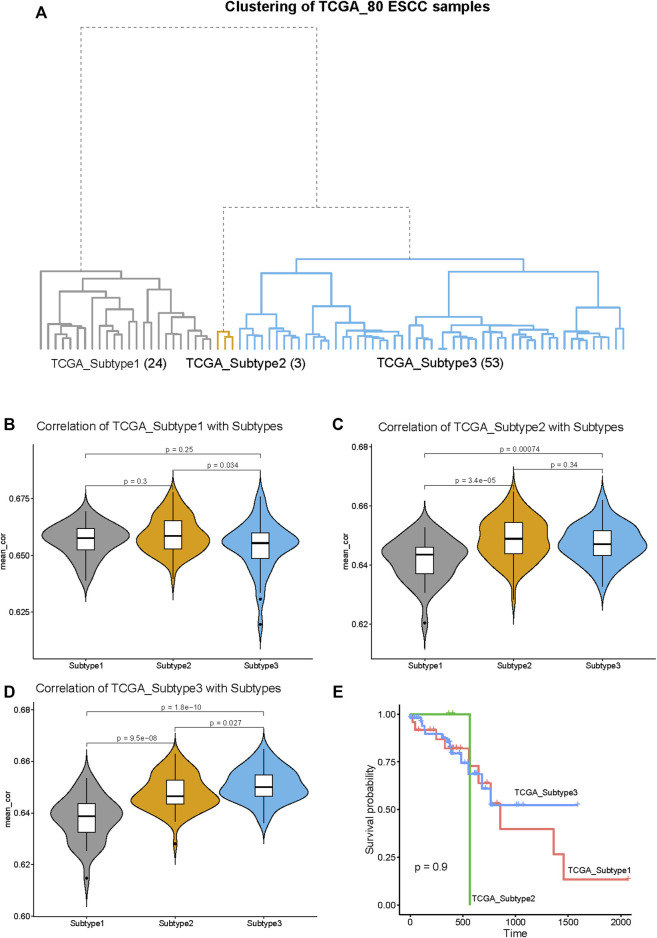
Validation of the generality of our ESCC Subtypes based on the RNA-seq data from TCGA database. **(A)** The hierarchical clustering tree of the 80 ESCC samples based on the RNA-seq data from TCGA database. The three subtypes are designated as: TCGA_Subtype1 (24 samples), TCGA_Subtype2 (3 samples), and TCGA_Subtype3 (53 samples). **(B)** The violin plot of the average correlation values between TCGA_Subtype1 and Subtypes (Subtype 1, Subtype 2 and Subtype 3). The average correlation values of TCGA_Subtype1 and Subtype 1 are significantly higher than TCGA_Subtype1 with Subtype 2/Subtype 3. **(C)** The violin plot of the average correlation values between TCGA_Subtype2 and Subtypes. The average correlation values of TCGA_Subtype2 and Subtype 2 are significantly higher than TCGA_Subtype2 with Subtype 1/Subtype 3. **(D)** The violin plot of the average correlation values between TCGA_Subtype3 and Subtypes. The average correlation values of TCGA_Subtype3 and Subtype 3 are significantly higher than TCGA_Subtype3 Subtype 1/Subtype 2. **(E)** Survival analysis between TCGA_Subtypes. Kaplan–Meier curves showing there is no significant difference between TCGA_Subtypes in survival.

To explore the generality of our classification, we clustered 80 ESCC tumor samples in the TCGA database and compared the obtained three subtypes with the three subtypes obtained by the 141 ESCC samples above. The 80 ESCC samples were clustered into three subtypes, named as TCGA_Subtype1 (24 samples), TCGA_Subtype2 (3 samples), TCGA_Subtype3 (53 samples) (Figure 8A). These three TCGA_Subtypes correspond one-to-one with the positions of the three Subtypes obtained by 141 ESCC samples on the clustering tree (Figure 1A, Figure 8A).

Through the correlation analysis between 80 TCGA ESCC samples and 141 GEO ESCC samples, it was found that the average correlation between subtypes located at the same position on the clustering tree is significantly higher than the average correlation between subtypes in different positions (Figures 8B–D). For example, the average correlation value between TCGA_Subtype3 and Subtype 3 is significantly higher than that TCGA_Subtype3 and Subtype 1 or Subtype 2 (Figure 8D). Therefore, our classification of ESCC into three subtypes by gene expression profiling is of general significance.

The survival analysis revealed no significant differences in survival between TCGA_Subtypes (Figure 8E). This means that the three ESCC subtypes we found may not have differences in survival.

## 4 Discussion

In this study, we integrated the gene expression profiles from different studies to a comparable level by correcting batch effects. In total, the gene expression profiles of paired tumor and normal samples from 141 patients with ESCC were included in this study. The 141 patients were divided into three subtypes based on their tumor sample gene expression profiles. Then, detailed characteristics of the three subtypes were described. The results of DGE analysis revealed that subtypes are different to each other. Next, we identified typical phenotype in each subtype; Wnt signaling pathway activation in Subtype 1, downregulation of glycogen metabolism in Subtype 2 and immunosuppression in Subtype 3. WGCNA revealed finer regulation of biological pathways in the three subtypes and revealed hub genes with important regulatory status. Moreover, we validated several hub genes were survival-related based on RNA-seq data from TCGA database. By comparing the survival-related-gene expression level in three subtypes, we suggested that the genes involved in immune-related biological processes or cell proliferation-related processes were responsible for the survival of these subtypes. Finally, in the RNA-seq dataset from TCGA database we verified that the three ESCC subtypes we found were of general significance.

The striking differences among the three ESCC subtypes were manifested in several aspects. For example, the degree of change in the expression levels of most genes between tumor and match normal tissue gradually increased from Subtype 1 to Subtype 3, especially genes with copy number variation (CNV) in ESCC genome. One potential explanation for this phenomenon is the increased overall tumor mutational burden (TMB, the total number of mutations in cancer cell DNA). Several studies have reported that copy number variations on ESCC genome were consistent with changes in gene expression levels (Hu et al., 2009; Shi et al., 2013). Liu et al. (2016) have found that subgroups of ESCC have significantly different somatic mutational burdens, such as subgroup1 (0.75 mutations per Mb) in their study, which showed fewest somatic mutational burden compared with subgroups1a (11.85 mutations per Mb) and subgroup2 (3.71 mutations per Mb). This is consistent with our conjecture that the three subtypes we identified appear to have differences in TMB. A high TMB is associated with poor prognosis (Owada-Ozaki et al., 2018; Hwang et al., 2019; Cui et al., 2020), and TMB has been demonstrated as a selection biomarker of immune checkpoint blockade (ICB) cancer therapy (Chan et al., 2019). In addition, some biological processes also exhibit progressively stronger association from Subtype 1 to Subtype 3, such as upregulation of the cell proliferation and dysfunction of endothelial cells in the results of co-expression modules. The progressively severe endothelial cell dysfunction from Subtype 1 to Subtype 3 means aggravated hypoxic environment and accelerated angiogenesis. Endothelial cells line the vascular systems and play important roles in tumorigenesis. The tumor microenvironment suffers from hypoxia, which will continuously stimulate blood vessel formation (Potente et al., 2011; Jing et al., 2019). These rapidly growing blood vessels are naturally differentiated from normal blood vessels, and tumor endothelial cells (TECs) exhibit different cell proliferation and migration ability compared with normal endothelial cells (NECs) (Hida et al., 2016). Accordingly, it is effective to subtyping ESCC from the perspective of transcriptome, which helps us to understand the molecular characteristics of ESCC more deeply and provide reference for precise treatment.

Subtype 1 was associated with the activation of Wnt signaling pathway, which will be a promising treatment target in ESCC. As an important pathway, Wnt signaling pathway activation has repeatedly been demonstrated in ESCC (Song et al., 2014; Sawada et al., 2016). Besides, Wnt signaling was reported to be inversely correlated with T cell infiltration in colorectal cancer, (Grasso et al., 2018), as we also found a lower T cell infiltration in subtype 1 than in other subtypes. The Wnt signaling pathway activation is associated with tumorigenesis and progression (Nusse and Varmus, 1992; Zhan et al., 2017; You et al., 2019); tumor proliferation and progression were inhibited by suppressing this pathway (Lee et al., 2012; You et al., 2019; Yu et al., 2021).

Significantly reduced glycogen metabolism in Subtype 2 may lead to glycogen accumulation in tumor tissue. Glycogen accumulation in tumor tissue is a distinguishing feature in various cancers and which promotes tumor development and maintenance (Iida et al., 2012; Maruggi et al., 2019; Xie et al., 2021). Accelerating glycogen metabolism can play a role in suppressing tumors, several enzymes involved in glycogen metabolism exert tumor-suppressive effects, including the glycogen debranching enzyme AGL and the kinase PhK β-subunit (PHKB) (Guin et al., 2016; Richmond et al., 2018; Yang et al., 2019; Liu et al., 2021). So, promoting glycogen metabolism may be a way to inhibit Subtype 2 ESCC.

Subtype 3 is characterized by immunosuppression, including downregulation of neutrophil degranulation and B/T cell related immune pathway. Assuming the downregulation of neutrophil degranulation was caused by the reduced total number of tumor-infiltrating neutrophils, we performed the immune cell infiltration analysis. The result confirmed decreased of neutrophils infiltration in subtype 3 compared to subtype 1 or 2, which possibly lead to downregulation of neutrophil degranulation. Similar down-stream pathways were identified in gene co-expression network. We found the downregulation of neutrophil chemotaxis in Subtype 3 based on the enrichment result of module 0. In view of the crucial role of neutrophils in the pathogenesis of cancer (Mantovani et al., 2011) and its position in the regulation of innate and adaptive immunity (Scapini et al., 2000; Tecchio et al., 2013; Uribe-Querol and Rosales, 2015; Jaillon et al., 2020), the reduction of infiltrating neutrophils may also be part of the reason why B- and T-cell immune-related processes are affected in subtype 3. Actually, several single-cell studies have demonstrated an immunosuppressive microenvironment in ESCC (Zheng et al., 2020; Dinh et al., 2021). Therefore, Subtype 3 is an immunosuppressive ESCC subtype, which also makes it most likely to benefit from immunotherapy. Wang et al. (2019) have found one immune-activate ESCC subtype by comparing two ESCC subtypes they have identified, this is significantly different from our Subtype 3. The difference may arise due to differences in analytical methods. They characterize the subtypes by making comparisons between the subtypes and we characterize the subtypes by comparing the tumor tissue to the normal tissue.

These nine survival-related genes are in key regulatory positions in the gene expression of the three subtypes; they are of great significance for the tumorigenesis and progression. Six of the nine survival-related genes (*CORO1A, CD180, SASH3, CD52, CD300A, and CD14*) were involved in the immune pathways, and were all were negatively correlated with survival, which is consistent with our understanding of the duality of immunity in tumors: immune has dual roles of suppressing and promoting cancer (Schreiber et al., 2011). *CD180* and *SASH3* are involved in the B cell-related immune pathway. The *SASH3*-encoded protein acts as a signaling protein in lymphocytes, and high SASH3 expression was associated with poor survival in cellular renal cell carcinoma (Yin et al., 2020). *CD52* and *CORO1A* are involved in regulating calcium homeostasis in immune cells. Calcium homeostasis is important for regulating the activation and function of macrophages, dendritic cells, and T cells (Zophel et al., 2020). *CD300A* and *CD14* are involved in neutrophil degranulation. CD14 is a key molecule in innate immunity activation, patients with bladder cancer with high CD14 levels may develop a proliferative tumor microenvironment (Cheah et al., 2015). The inhibitory receptor protein CD300A is found on leukocytes and is involved in the immune response signaling pathways; the interaction between CD300A and phosphatidylserine can inhibit the killing effect of natural killer (NK) cells on tumor cells (Lankry et al., 2013). In addition to these six survival-related genes involved in immune-related processes, the remaining three survival-related genes are involved in other biological processes. The mechanism of DUSP1 in organisms is highly complex: it is a transcriptional target of tumor suppressor p53 (Li et al., 2003) and also responds to various cellular stress conditions (Keyse and Emslie, 1992). In invasive ovarian cancer, *DUSP1* expression was significantly associated with shorter progression-free survival (*p* = 0.019) (Denkert et al., 2002). *KIF14* and *MCM2* are involved in numerous biological pathways, including G2–M transition, mitotic nuclear division, and DNA replication. *MCM2* has a coregulatory role in ESCC progression and may have core roles during the pathogenesis of ESCC (Wang et al., 2018). MCMs increase genome duplication robustness by restraining the speed at which eukaryotic cells replicate their DNA, where a minor reduction in MCM levels destabilizes the genome and predisposes to increased incidence of tumor formation (Sedlackova et al., 2020). Genes can influence the overall performance of transcriptional profile through different regulatory strategies (Wang et al., 2021). The roles of these nine survival-related genes in ESCC need further explorations and may become potential targets for ESCC therapy in the future.

There are some limitations of this study. The clinical information of these 141 ESCC samples was not comprehensive enough to interpret existing findings in combination with more clinical information. In addition, the genes with prognostic value have not been validated in other independent cohorts. Finally, due to the limited laboratory conditions, some of our results have not been verified in cell experiments.

In conclusion, we have identified three subtypes of ESCC by large-scale gene expression profiling of tumor tissues. Through in-depth exploration of these three subtypes, we have characterized the three subtypes from multiple perspectives and discovered some new potential targets that may be effective in the treatment of ESCC. Taken together, our results suggest that distinct ESCC subtypes defined using transcriptomes may exhibit better responses to specific targeted therapies. Actually, the effectiveness of these targets needs further exploration and verification. Our findings have deepened our understanding of the molecular characteristics of ESCC and provided some references for future clinical treatment research.

## Data Availability

The original contributions presented in the study are included in the article/Supplementary Material, further inquiries can be directed to the corresponding author.
