# Supplementary material for: Molecular subtyping of esophageal squamous cell carcinoma by large-scale transcriptional profiling: Characterization, therapeutic targets, and prognostic value
Source: Front Genet. 2022 Nov 8;13:1033214. doi: 10.3389/fgene.2022.1033214 (PMC9678939; doi:10.3389/fgene.2022.1033214)
Supplement: Supplementary file 3 [file DataSheet1.PDF]

**Supplementary figure legends:**

**Supplementary Figure 1.** The expression profile of the seven datasets after combined. (A) The PCA (principal components analysis) plot grouped by batch before batch correction. (B) The PCA plot grouped by batch after batch correction. (C) The boxplot of the combined data before batch correction. (D) The boxplot of the combined data after batch correction. (E) The PCA plot grouped by normal and tumor after batch correction. (F) The PCA plot grouped by tumor and normal after removing three outliers (tumor and paired normal samples) (sample size = 282).

**Supplementary Figure 2.** The hierarchical clustering tree of 141 ESCC samples that labeled by different groups. (A) The hierarchical clustering of the 141 ESCC samples labeled by batch. (B) The hierarchical clustering of the 141 ESCC samples labeled by K-means_Subtype.

**Supplementary Figure 3.** The volcano plots of differential expressed genes (DEGs) in three subtypes. Subtypes 1, 2, and 3 had 83, 376, and 743 total DEGs, respectively.

**Supplementary Figure 4.** The profiles of 18 DEGs that were specific to Subtype 1 or 2 rather than Subtype 3. (A) The log2FC of 18 genes in three subtypes. (B) The 16 upregulated DEGs among these 18 genes are enriched in the immune-related pathways.

**Supplementary Figure 5.** Immune cell infiltration scores in tumor tissues of three ESCC subtypes. There are fewer infiltrating T cells in Subtype 1 and less neutrophil infiltration in Subtype 3 tumor tissues.

**Supplementary Figure 6.** Enrichment of biological pathways in module 2.

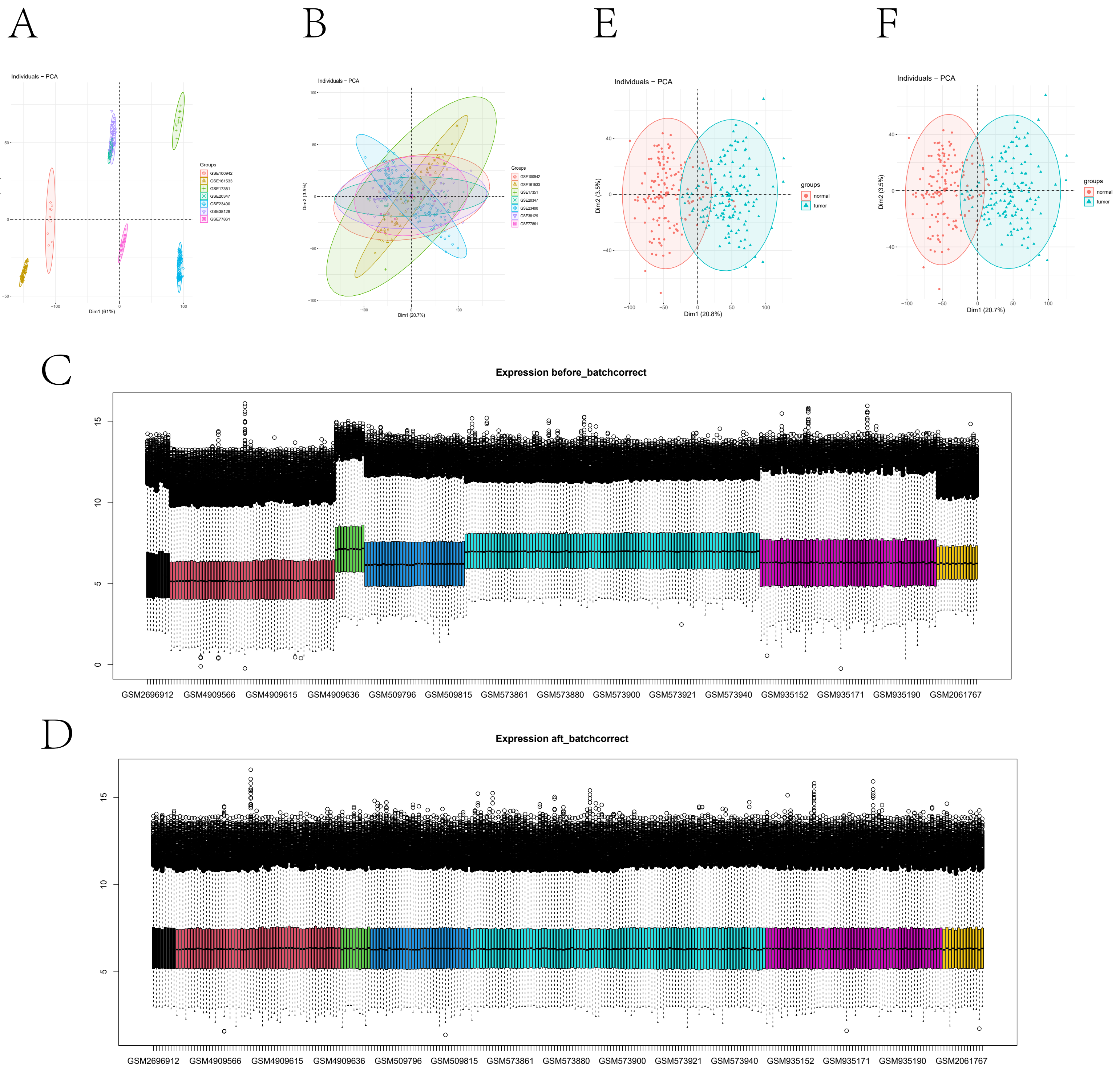


Supplementary Figure 1

A

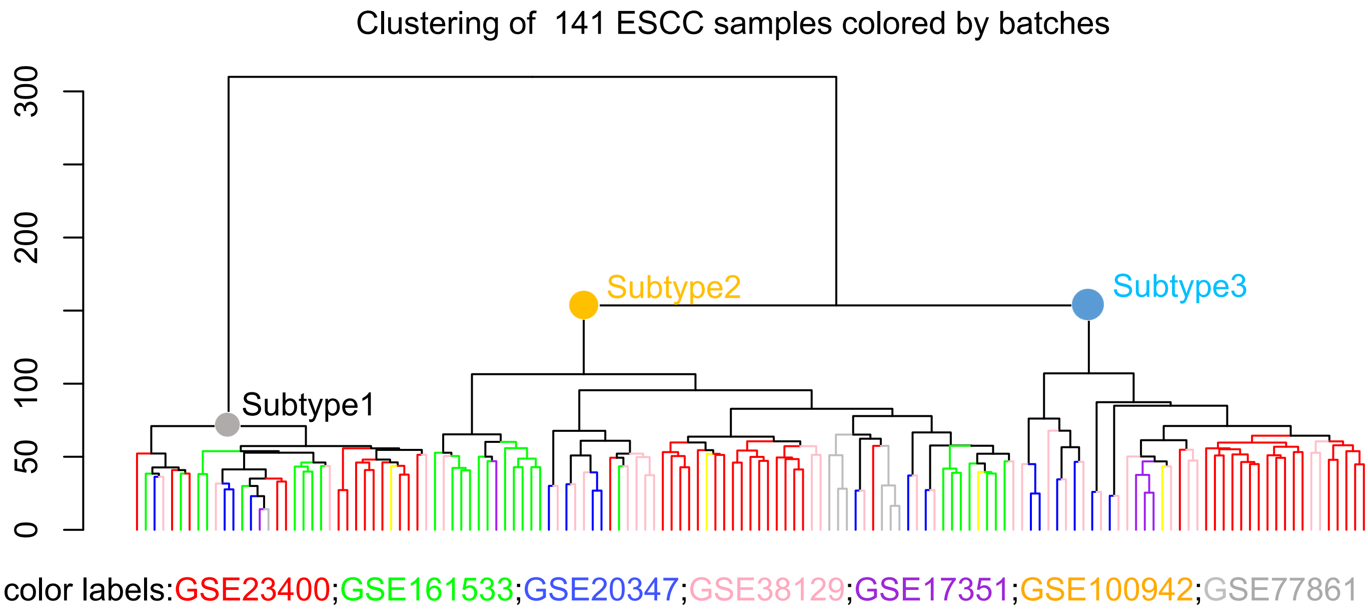


B

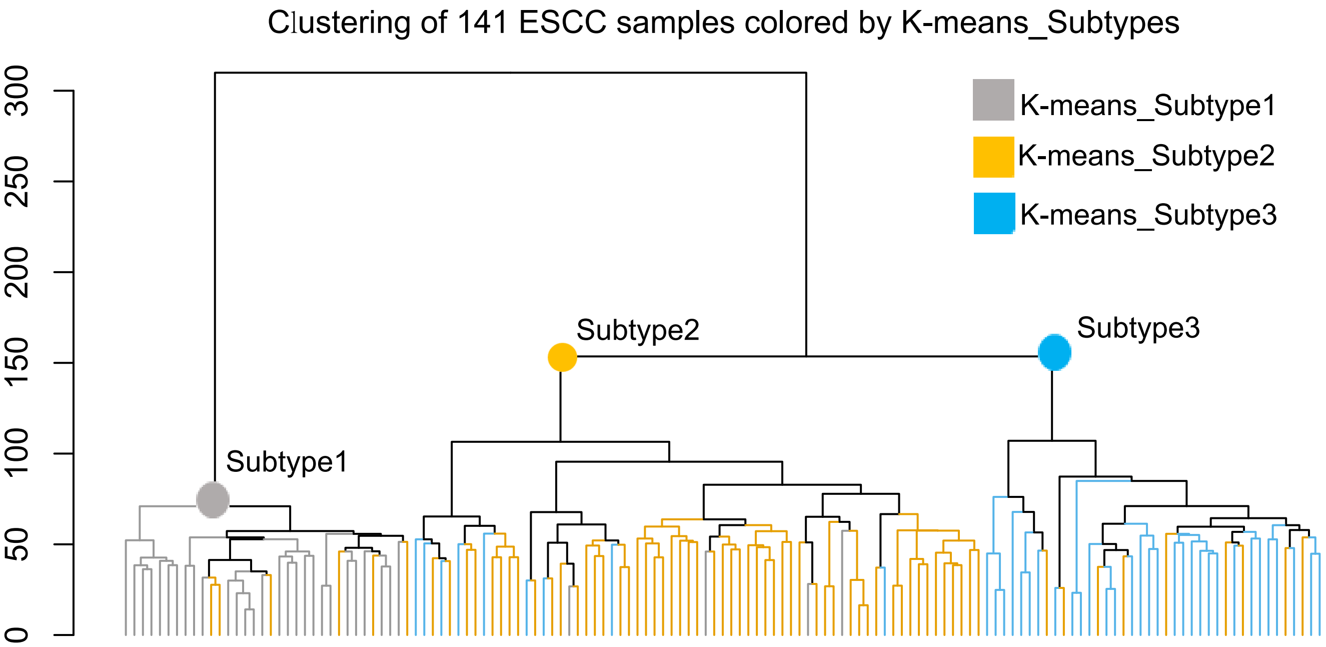


Supplementary Figure 2

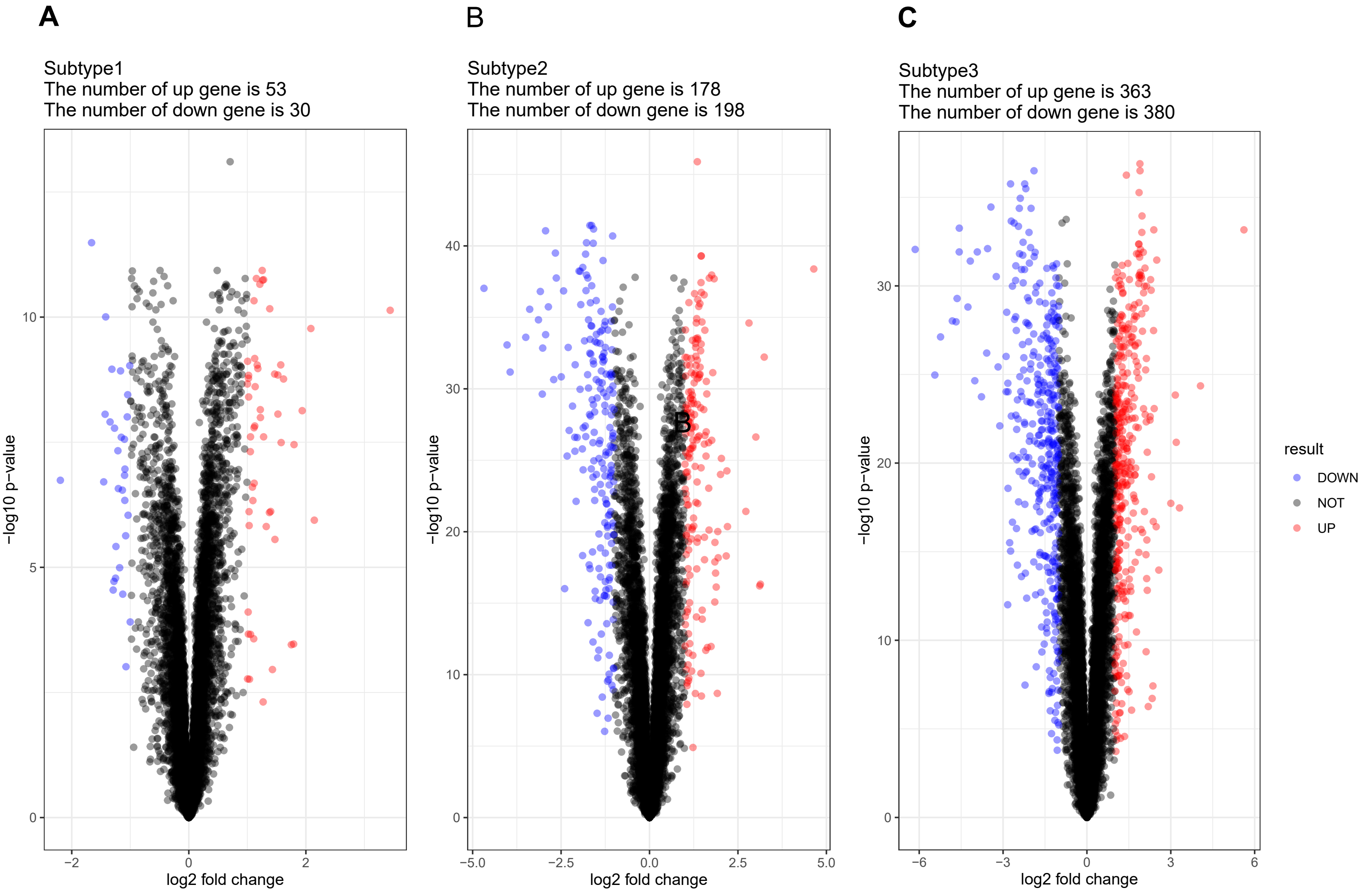


Supplementary Figure 3

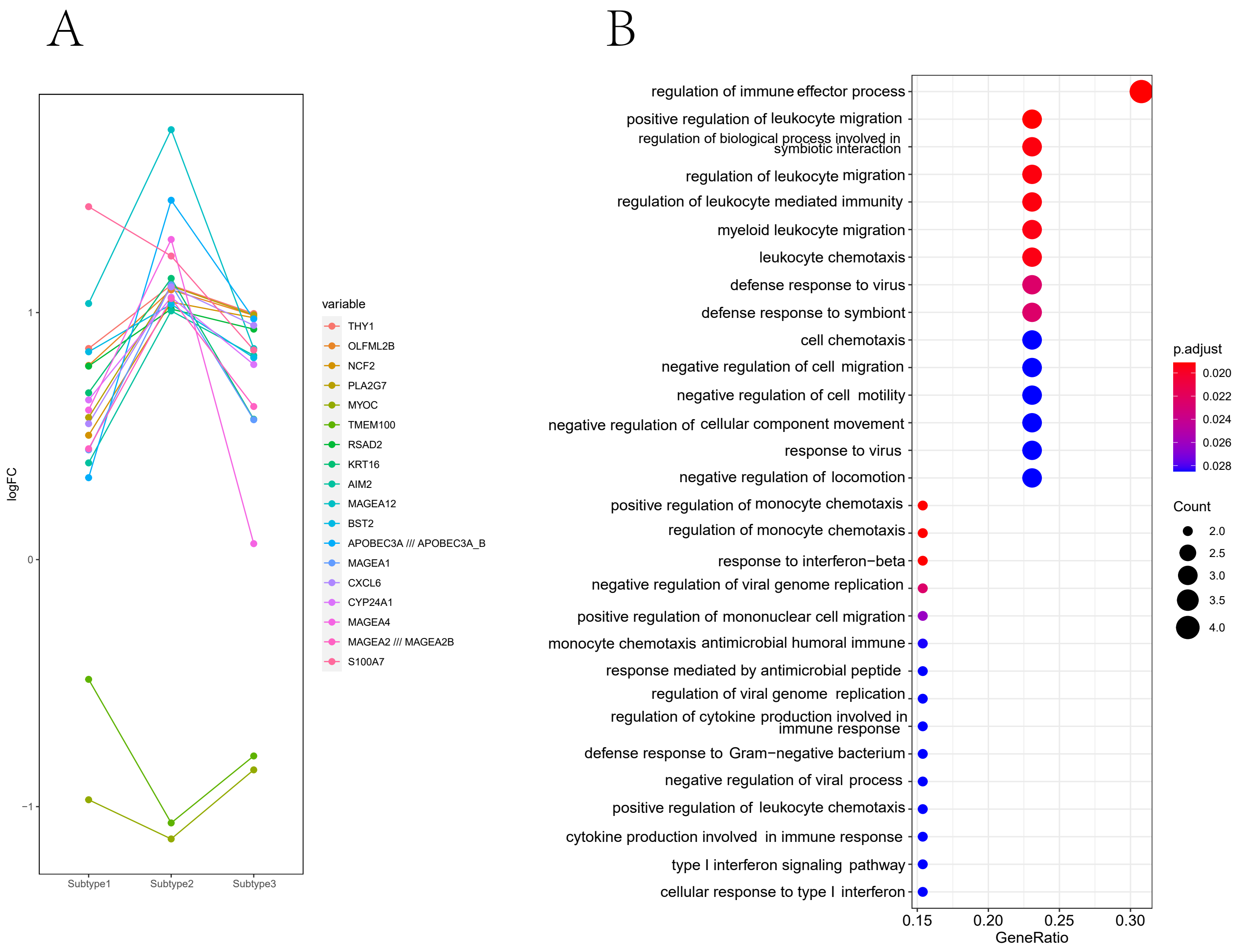


Supplementary Figure 4

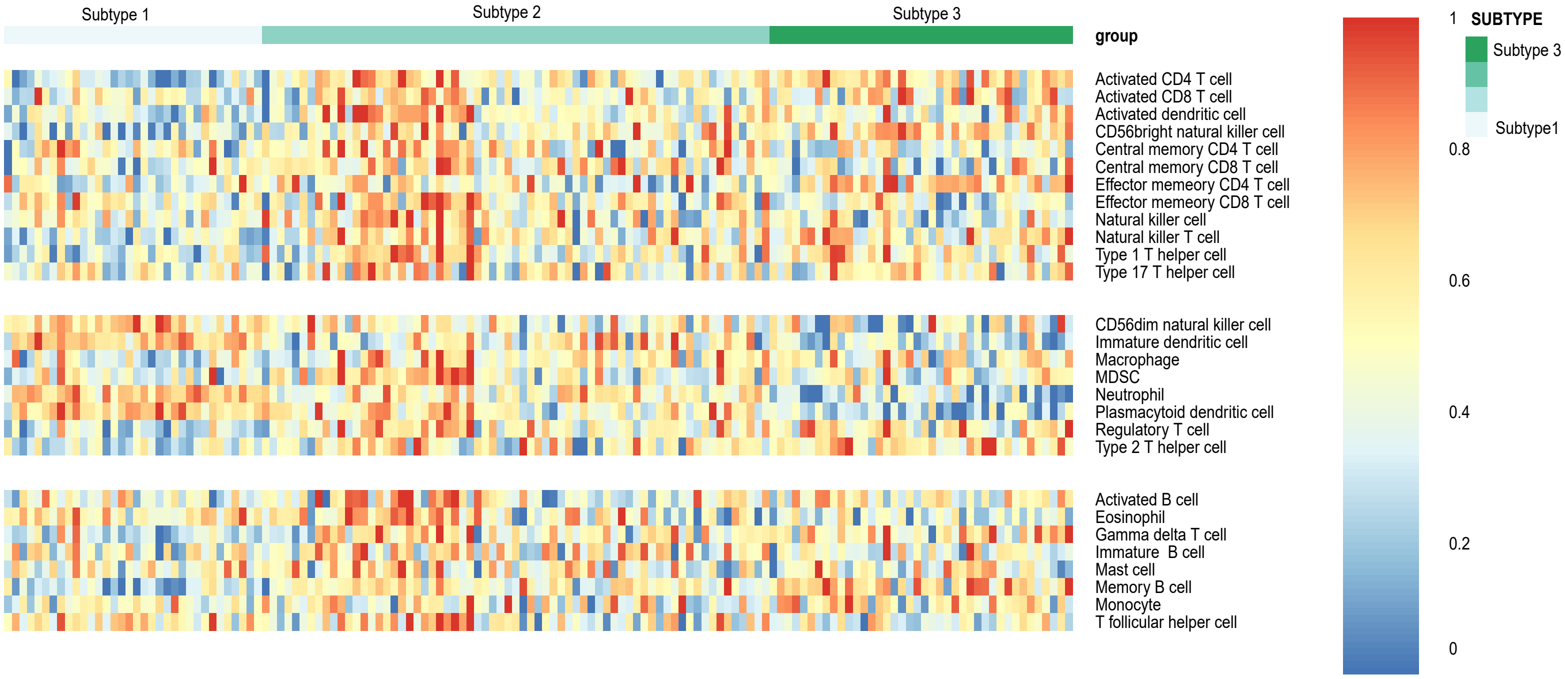


Supplementary Figure 5

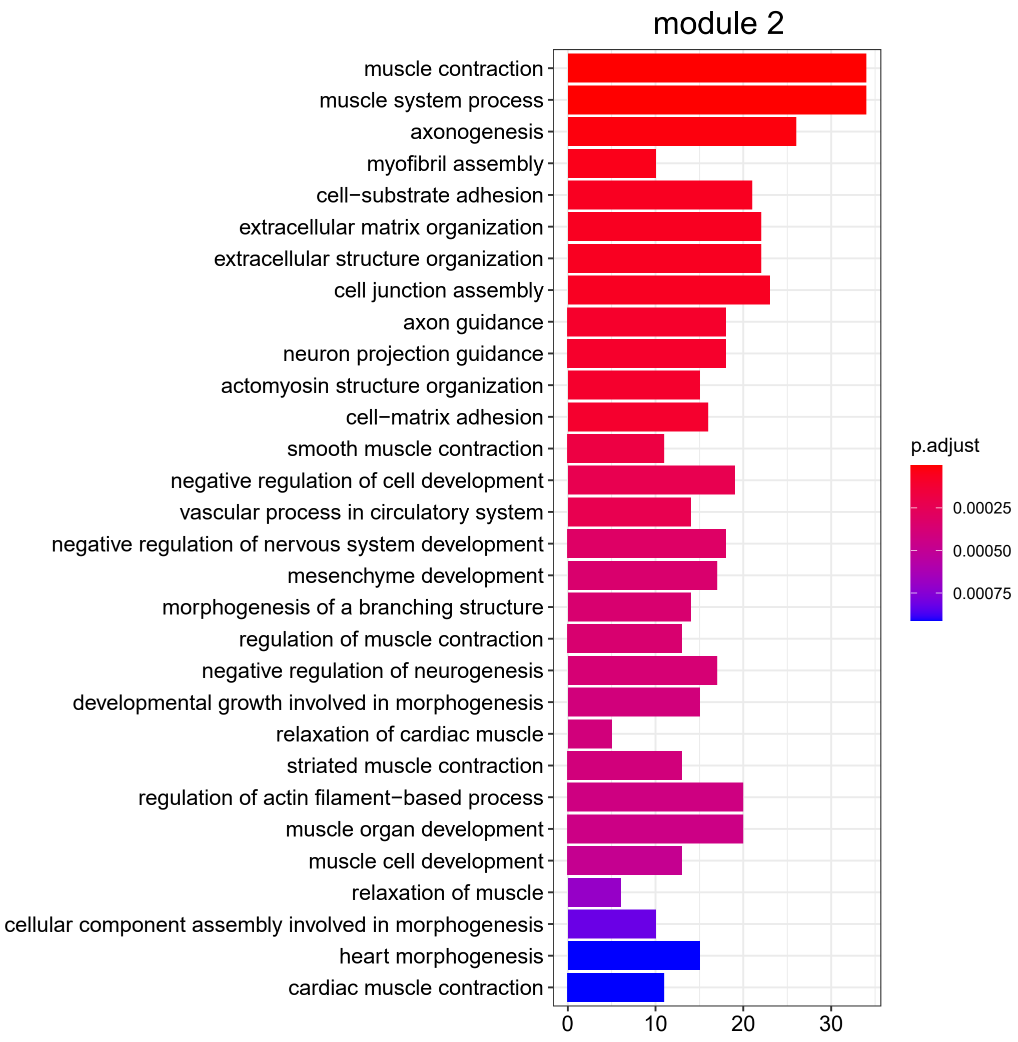


Supplementary Figure 6
